# Allergic diseases in the elderly population: a single-center experience

**DOI:** 10.3906/sag-2104-55

**Published:** 2021-10-21

**Authors:** Eray YILDIZ, Fatih ÇÖLKESEN, Şevket ARSLAN, Recep EVCEN, Filiz SADİ AYKAN, Mehmet KILINÇ, Gökhan AYTEKİN

**Affiliations:** 1 Department of Immunology and Allergy, Meram Faculty of Medicine, Necmettin Erbakan University, Konya Turkey; 2 Department of Immunology and Allergy, Konya City Hospital, Konya Turkey

**Keywords:** Elderly, allergic diseases, allergic rhinitis, drug hypersensitivity reactions, hymenoptera venom allergy, anaphylaxis

## Abstract

**Background/aim:**

Although allergic diseases are generally considered to be diseases of childhood and youth, the first symptoms of allergic diseases can be seen in old age sometimes. The aim of this study was to determine the prevalence and characteristics of allergic diseases in the elderly population admitted to the allergy unit on an outpatient basis.

**Materials and methods:**

The files of the patients who applied to our clinic’s allergy unit during the 8-year period were retrospectively analyzed. The data of patients aged ≥ 65 years were obtained from the files of our allergy unit archive.

**Results:**

A total of 1272 patients aged ≥ 65 years old were included in the study. The mean age was 70 years (range: 65–97 years). Most of the patients were female (n = 704, 55.3%). Of the patients, 887 (69.8%) presented with cutaneous symptoms, and urticaria was identified in 500 of them (56.3%). Drug hypersensitivity reactions were detected in 175 (13.7%) patients. A total of 71 (5.6%) patients had asthma, 65 (5.1%) had anaphylaxis, 48 (3.8%) had allergic rhinitis, 24 (1.9%) had hymenoptera venom allergy, and 18 (1.4%) had food allergies.Atopy history (OR = 2.323, 95% CI = 1.590–3.393, p < 0.001) and comorbidity (OR = 1.631, 95% CI = 1.050–2.533, p = 0.029) were found to be risk factors for drug hypersensitivity reactions. Male sex (OR = 3.462, 95% CI = 1.097–10.933, p = 0.034) and atopy history (OR = 14.877, 95% CI = 6.081–36.393, p < 0.001) were found to be risk factors for hymenoptera venom allergy.

**Conclusion:**

Diagnosis becomes difficult due to the perception that allergic diseases mainly affect young people. Clinical symptoms are not evident in the elderly and age-related difficulties are encountered in diagnostic tests. There is a need to develop specific guidelines for the diagnosis of allergic diseases in the elderly.

## 1. Introduction

The number of elderly people around the world is growing, and accordingly, life expectancy is gradually increasing. Increased life expectancy leads to an increase in the incidence of many pathologies, including allergic diseases [1].

Although allergic diseases are generally considered to be diseases of childhood and youth, they usually continue until old age, and the first symptoms of allergic diseases can sometimes be seen in old age [2]. Nonetheless, there is insufficient literature data on the diagnosis, course, and treatment management of allergic diseases in the elderly, because the symptoms developing in these patients are considered to be a part of the current multiple disease manifestation, and allergic disease symptoms often improve spontaneously without being noticed [3,4].

We observed an increase in the frequency of the elderly population who applied to our clinic’s allergy unit on an outpatient basis. With this study, we aimed to determine the prevalence and characteristics of allergic diseases in the elderly population.

## 2. Materials and methods

### 2.1. Study design

The files of the patients who applied to our clinic’s allergy unit between June 2012 and December 2020 were retrospectively scanned. The data of patients aged ≥ 65 years were obtained from the files of our allergy unit archive.

The study was approved by the local ethics committee of the Necmettin Erbakan University Meram Faculty of Medicine (Decision no: 2020/2594).

### 2.2. Data collection

The patients age, sex, atopy history, comorbidities, complaints, objective physical examination findings, and results of allergen-specific immunoglobulin (Ig) E, serum tryptase, and skin tests were obtained from medical records. Skin tests performed on patients included skin prick testing (SPT) for inhalant and food allergens and skin patch testing (PT) for contact allergens.

#### 2.2.1. Skin prick test

The SPT included inhaled allergens (Allergopharma, Germany) (grasses mix [dactylis, festuca, lolium, phleum, poa], tree mix [alnus, betula, corylus], weed mix [artemisia, chenopodium, plantago, salsola], latex, canis familiaris, felis domesticus, dermatophagoides mix, alternaria alternata, and blattella germanica) and food allergens (Allergopharma, Germany) (rye flour, wheat flour, barley flour, oat flour, soy, sesame, egg white, egg yolk, chicken meat, cow milk, almond, hazelnut, walnut, celery, peach, rice, peanut, carrot, cow meat, and cashew).

Histamine (10 mg/mL) was used as a positive control, and 0.9% NaCl was used as a negative control.

#### 2.2.2. Patch test

TRUE (thin layer rapid use epicutaneous) test ready-to-use PT panels (Allerderm, Denmark) were used. The readings were taken at 48 and 72 h. The results were evaluated as negative, positive (+, ++, +++), and irritant reactions.

#### 2.2.3. Venom-specific Ig E and tryptase

Venom-specific (Apis mellifera, Vespula spp.) Ig E and tryptase levels were detected by immunoassay (UniCAP 100E, Phadia, Uppsala, Sweden). Venom-specific Ig E levels below 0.35 kU/L were considered negative. The normal tryptase level was below 11.4 µg/L.

### 2.3. Statistical analysis

The data obtained in the study were analyzed using SPSS for Windows version 22.0 software. Continuous variables were expressed as mean ± standard deviation or median (min-max), and categorical variables as numbers with percentages. Univariate logistic regression analyzes were used to identify risk factors in allergic diseases. p values < 0.05 were considered to be statistically significant.

## 3. Results

A total of 1272 patients aged ≥ 65 years old were included in the study. The mean age was 70 years (range: 65–97 years), and most of the patients were female (n = 704, 55.3%). A total of 1006 (79.1%) had at least one comorbid disease. The most common comorbid diseases were cardiovascular system diseases (n = 812, 63.8%), diabetes mellitus (n = 323, 23.4%) and respiratory system diseases (n = 170, 13.4%) (Table 1).

**Table 1:** General characteristics of patients aged ≥ 65 years.

	n (%)
Total	1272
Age, median (min-max)	70 (65–97)
Sex	
Female	704 (55.3)
Male	568 (44.7)
Atopy	193 (15.2)
Comorbidity	1006 (79.1)
Cardiovascular diseases	812 (63.8)
Diabetes mellitus	323 (25.4)
Respiratory diseases	170 (13.4)
Thyroid diseases	110 (8.6)
Renal-urinary system diseases	96 (7.5)
Neuropsychiatric diseases	88 (6.9)
Malignant diseases	49 (3.9)
Rheumatological diseases	28 (2.2)
Gastrointestinal-liver diseases	27 (2.1)
Autoimmune diseases	7 (0.6)
Presentation forms	
Cutaneous symptoms	887 (69.8)
Rhinitis symptoms	155 (12.2)
Respiratory symptoms	144 (11.3)
Anaphylaxis	65 (5.1)
Conjunctivitis symptoms	21 (1.6)

### 3.1. Cutaneous allergic conditions

A total of 887 (69.8%) patients presented with cutaneous symptoms. The most common cause was urticaria in 500 (56.3%) of these patients, xerosis in 137 (15.4%), and systemic itching in 89 (10%). The rarest cause in patients presenting with cutaneous symptoms was atopic dermatitis (AD), which was seen in 13 (1.5%) patients (Table 2).

**Table 2:** Characteristics of patients presenting with cutaneous symptoms.

	n (%)
Total	887 (69.8)
Urticaria, without angioedema1	427 (48.1)
Idiopathic	327 (76.6)
Drug	87 (20.4)
Antibiotics	27 (6.3)
NSAIDs	25 (5.9)
Others 2	35 (8.2)
Food	12 (2.8)
Venom	1 (0.2)
Urticaria, with angioedema1	73 (8.2)
Idiopathic	48 (65.8)
Drug	20 (27.4)
Antibiotics	13 (17.8)
NSAIDs	5 (6.8)
Others	2 (2.8)
Food	3 (4.1)
Venom	2 (2.7)
Angioedema, without urticaria3	58 (6.5)
ACE inhibitors4	35 (60.4)
Idiopathic	9 (15.5)
NSAIDs	8 (13.9)
Other drugs	3 (5.1)
Food	1 (1.7)
Venom	1 (1.7)
Acquired	1 (1.7)
Xerosis	137 (15.4)
Systemic diseases5	89 (10)
Allergic contact dermatitis6	39 (4.5)
Atopic dermatitis7	13 (1.5)
Other skin diseases8	51 (5.8)

NSAIDs: Nonsteroidal antiinflammatory drugs, ACE: Angiotensin converting enzyme.1 Sex, female (n = 295, 59%), male (n = 205, 41%), atopy (n = 82, 16.4%), comorbidity (n = 398, 79.6%).2 Cardiovascular drugs (mainly ACE inhibitors and beta-blockers) (n = 8), chemotherapeutics (n = 8), allopurinol (n = 5), local anesthetics (n = 4), contrast agents (n = 3), proton pump inhibitors (n = 2), insulin (n = 2), methimazole (n = 1), pregabalin (n = 1), infliximab (n =1)3 Sex, female (n = 32, 55%), male (n = 26, 45%), atopy (n = 82, 16.4%), comorbidity (n = 12, 21%).4 ACE inhibitor-induced angioedema; 4.7% (n = 35/738). Sex, female (n = 20, 57%), male (n = 15, 43%), atopy (n = 7, 35%), comorbidity (n = 35, 100%).5 Systemic diseases; liver diseases, kidney diseases, hematologic diseases, endocrine disorders, neurologic diseases, paraneoplasic pruritus, infectious diseases.6 Ten patients who developed gingivitis and stomatitis > 24 h after dental prosthesis application were accepted as contact allergy to dental metals. Sex, female (n = 20, 51%), male (n = 19, 49%), atopy (n = 3, 7.7%), comorbidity (n = 29, 74%).7 Sex, male: 77% (n = 10), female: 23% (n = 3), atopy (n = 4, 31%), comorbidity (n = 11, 85%).8 Other skin diseases; all skin diseases other than the above diagnoses (most common scabies and bullous pemphigoid).

### 3.2. Drug hypersensitivity reactions

Drug hypersensitivity reactions (DHRs) were observed in a total of 175 (13.7%) patients. Depending on the time of their emergence, DHRs were divided into two categories: immediate (1–6 h) and delayed (> 6 h) [5]. One hundred sixty-three (93.1%) of the patients presented with immediate DHRs and 12 (6.9%) with delayed DHRs. The most common clinical presentation was urticaria in 107 (65.7%) of the patients presenting with immediate type DHRs, Stevens–Johnson syndrome (SJS), and toxic epidermal necrolysis (TEN) in six (50%) of the patients presenting with delayed type DHRs. The most common cause was nonsteroidal antiinflammatory drugs (NSAIDs) in 67 (41.1%) of the patients presenting with immediate type DHR, and allopurinol in four (33.3%) of the patients presenting with delayed type DHRs (Table 3).

**Table 3:** Characteristics of patients with drug hypersensitivity reactions.

	n (%)
Total	175 (13.7)
Sex	
Female	99 (57)
Male	76 (43)
Atopy	46 (26)
Comorbidity	149 (85)
Immediate type	163 (93.1)
Urticaria, without angioedema	87 (53.4)
Antibiotics1	27 (31)
NSAIDs	25 (28.7)
Cardiovascular drugs	8 (9.3)
Chemotherapeutics	8 (9.3)
Allopurinol	5 (5.7)
Local anesthetics	4 (4.6)
Contrast agents	3 (3.4)
Others	7 (8.0)
Anaphylaxis	45 (27.6)
NSAIDs	21 (46.7)
Antibiotics2	19 (42.2)
Others3	5 (11.1)
Urticaria, with angioedema	20 (12.3)
NSAIDs	13 (65)
Antibiotics1	5 (25)
Others	2 (10)
Angioedema, without urticaria	11 (6.7)
NSAIDs	8 (72.7)
Others	3 (27.3)
Delayed type	12 (6.9)
SJS/TEN	6 (50)
Allopurinol	2 (33.2)
Carbamazepine	2 (33.2)
NSAIDs	1 (16.6)
Antibiotics	1 (16.6)
DRESS	3 (25)
Allopurinol	2 (66.7)
Carbamazepine	1 (33.3)
Allergic contact dermatitis	2 (16.7)
NSAIDs	2 (100)
FDE	1 (8.3)
NSAIDs	1 (100)

NSAIDs: Nonsteroidal antiinflammatory drugs, SJS: Stevens–Johnson syndrome, TEN: Toxic epidermal necrolysis, DRESS: Drug reaction with eosinophilia and systemic syndrome, FDE: Fixed drug eruption.1 Beta lactams (54%), quinolones (25%).2 Beta lactams (84.2%).3 Proton pump inhibitors (n = 2), local anesthetics (n = 1), vitamin B12 (n = 1), ranitidine (n = 1).

### 3.3. Allergic rhinitis and asthma

Allergic rhinitis (AR) was identified in 48 (3.8%) patients. Twenty-nine (60%) patients with AR had sensitivity to two or more allergens. The most common allergen was pollen (n = 38, 81.4%). Ten (21%) of the patients with AR had asthma. Furthermore, 21 (1.6%) of the patients presented with symptoms of conjunctivitis, the majority of which were associated with rhinitis. Seventy-one of the patients (5.6%) were diagnosed with asthma (Table 4).

**Table 4:** Characteristics of patients presenting with rhinitis and respiratory symptoms.

	n (%)
Rhinitis symptoms	
Total	155 (12.2)
Nonallergic rhinitis	107 (8.4)
Allergic rhinitis	48 (3.8)
Age	
65–74 years	44 (91)
≥ 75 years	4 (9)
Sex	
Male	25 (52)
Female	23 (48)
Comorbidity	35 (73)
Allergens	
Pollens1	38 (81.4)
Mites	30 (62.6)
Blatella germanica	7 (14.6)
Cat	4 (8.3)
Alternaria alternata	4 (8.3)
Latex	1 (2.1)
Respiratory symptoms	
Total	144 (11.4)
Asthma	71 (5.6)
Sex	
Female	54 (76)
Male	17 (24)
Comorbidity	53 (75)
Atopy	19 (27)
Spirometry	17 (24)
Allergens	
Pollens 2	7 (61.6)
Mites	5 (38.5)
Alternaria alternata	2 (15.4)
Cat	1 (7.7)
Other respiratory conditions	73 (5.8)
ACE inhibitor associated cough	17 (23.3)
Chronic obstructive pulmonary disease	12 (16.4)
Gastroesophageal reflux disease	12 (16.4)
Heart failure	11 (15.1)
Pneumonia	8 (11)
Post nasal discharge	8 (11)
Others	5 (6.8)

ACE: Angiotensin converting enzyme.1 Weed pollens (44.7%), grass pollens (39.5%).2 Weed pollens (57%).

### 3.4. Anaphylaxis

The diagnosis of anaphylaxis was made according to the World Allergy Organization guidance [6]. Sixty-five (5.1%) of the patients presented with anaphylaxis. The cause of anaphylaxis was medication in 45 (69.2%) patients, venom in 19 (29.2%) patients, and food in one (1.6%) patient (Table 5).

**Table 5:** Characteristics of patients with anaphylaxis.

	n (%)
Total	65 (5.1)
Sex	
Female	34 (52)
Male	31 (48)
Atopy	36 (55)
Comorbidity	50 (77)
Drugs	45 (69.2)
NSAIDs	21 (46.7)
Antibiotics	19 (42.2)
Beta lactams	16 (84.2)
Others	3 (15.8)
Others	5 (10.1)
Venom	19 (29.2)
Vespula spp.	8 (42.1)
Apis mellifera	7 (36.8)
Apis mellifera + Vespula spp.	4 (21.1)
Food	1 (1.6)

NSAIDs: Nonsteroidal antiinflammatory drugs.

### 3.5. Hymenoptera venom allergy

Twenty-four (1.9%) patients were observed to have hymenoptera venom allergy (HVA), and 11 (46%) had a reaction with Vespula spp. Twenty-two (91.7%) of the patients had systemic reactions, 10 (45.5%) of which were grade II [7] (Table 6).

**Table 6:** Characteristics of patients with hymenoptera venom allergy.

	n (%)
Total	24 (1.9)
Sex	
Male	20 (83)
Female	4 (17)
Atopy	17 (71)
Comorbidity	18 (75)
Clinic	
Systemic reaction	22 (91.7)
Grade I	3 (13.6)
Grade II	10 (45.5)
Grade III	7 (31.8)
Grade IV	2 (9.1)
Large local reaction	2 (8.3)
Insects	
Vespula spp.	11 (46)
Apis mellifera	8 (33)
Apis mellifera + Vespula spp.	5 (21)
Patients admitted to the emergency room	19 (79)
Patients carrying adrenaline auto-injectors	19 (79)
Patients started immunotherapy	2 (8.5)
Beekeepers	9 (37.5)
Tryptase	
> 11.4 µg/L	2 (8.3)

### 3.6. Food allergy

Eighteen patients (1.4%) were observed to have food allergy (FA). In 14 (78%) of these patients, urticaria was the most common clinical presentation. The most common allergen was peanuts, observed in five (33%) patients (Table 7).

**Table 7:** Characteristics of patients with food allergy.

	n (%)
Total	18 (1.4)
Sex	
Female	11 (61)
Male	7 (39)
Atopy	9 (50)
Comorbidity	15 (83)
Clinic	
Urticaria, without angioedema	12 (67)
Urticaria, with angioedema	2 (11)
Rhinitis	2 (11)
Angioedema, without urticaria	1 (5.5)
Anaphylaxis1	1 (5.5)
Food	
Peanut	5 (33)
Egg	4 (22)
Soy	2 (11)
Apricot 2	1 (5.6)
Peach 2	1 (5.6)
Others 3	6 (33.5)

1 Peanut.2 Apricot-birch and peach-mugwort cross-sensitivity.3 Hazelnut (n = 1), apple (n = 1), strawberry (n = 1), wheat flour (n = 1), fish (n = 1), lobster (n = 1).

### 3.7. Skin prick test

A total of 787 (62%) patients underwent SPT. The result was positive in 110 (14%) patients. Of the patients with positivity for the inhalant allergens, 65 (59.1%) were detected as sensitive to pollen and 63 (57.3%) to mites. Among patients with food allergen positivity, the most common allergens identified were egg in five patients (4.5%), peanut in four (3.6%), and soy in four (3.6%) (Table 8).

**Table 8:** Results of skin test in patients.

	n (%)
Total	110 (14)
Inhalant allergens	86 (78.2)
Pollens	65 (59.1)
Weed pollens	35 (53.8)
Grass pollens	21 (32.3)
Tree pollens	9 (13.9)
Mites	63 (57.3)
Blatella	10 (9.1)
Alternaria	9 (8.2)
Cat and dog epithelium	6 (5.4)
Latex	2 (1.8)
Food allergens	20 (18.2)
Egg	5 (4.5)
Peanut	4 (3.6)
Soy	4 (3.6)
Cocoa 1	3 (2.7)
Others	10 (9)
Inhalant allergens + Food allergens	4 (3.6)

1 Cocoa-nickel cross-sensitivity was detected in three patients.

### 3.8. Risk factors for allergic diseases in the elderly

In the logistic regression analysis, female sex (Odds ratio [OR] = 0.760, 95% Confidence interval [CI] = 0.605–0.955, p = 0.019) was found to be a risk factor for urticaria, male sex (OR = 4.141, 95% CI = 1.134–15.120, p = 0.031) for AD. Atopy history (OR = 2.323, 95% CI = 1.590–3.393, p < 0.001) and comorbidity (OR = 1.631, 95% CI = 1.050–2.533, p = 0.029) were found to be risk factors for DHRs. Male sex (OR = 3.462, 95% CI = 1.097–10.933, p = 0.034) and atopy history (OR = 14.877, 95% CI = 6.081–36.393, p < 0.001) were found to be risk factors for HVA (Table 9).

**Table 9:** Logistic regression analysis results of risk factors for allergic diseases in the elderly.

	Comorbidity		Atopy		Sex	
	OR (95% CI)	p	OR (95% CI)	p	OR (95% CI)	p
Urticaria, with and without angioedema	1.059 (0.802–1.399)	0.686	0.950 (0.692–1.304)	0.752	0.760 (0.605–0.955)	0.019
Angioedema, without urticaria	1.904 (0.852–4.253)	0.116	1.570 (0.813–3.030)	0.179	1.063 (0.621–1.820)	0.822
Allergic contact dermatitis	0.764 (0.367–1.587)	0.470	0.460 (0.140–1.510)	0.201	1.168 (0.617–2.211)	0.633
Atopic dermatitis	1.482 (0.326–6.726)	0.610	2.530 (0.771–8.298)	0.126	4.141 (1.134–15.120)	0.031
Drug hypersensitivity reactions	1.631 (1.050–2.533)	0.029	2.323 (1.590–3.393)	< 0.001	0.934 (0.677–1.288)	0.676
Allergic rhinitis	0.705 (0.368–1.352)	0.293	3.287 (1.781–6.067)	< 0.001	1.345 (0.755–2.396)	0.314
Asthma	0.770 (0.443–1.338)	0.354	1.729 (0.968–3.086)	0.064	0.397 (0.230–0.685)	0.001
Anaphylaxis	0.888 (0.491–1.608)	0.696	8.363 (4.986–14.028)	< 0.001	1.126 (0.683–1.856)	0.641
Hymenoptera venom allergy	0.802 (0.315–2.040)	0.643	14.877 (6.081–36.393)	< 0.001	3.462 (1.097–10.933)	0.0341
Food allergy	1.346 (0.387–4.684)	0.640	5.852 (2.293–14.939)	< 0.001	1.280 (0.493–3.324)	0.612

OR: Odds ratio, CI: Confidence interval.1 Beekeepers excluded.

## 4. Discussion

### 4.1. Cutaneous allergic conditions

Itching is a common problem in the elderly; however, a wide range of differential diagnoses and the presence of polypharmacy and comorbidities makes its etiology difficult to determine [8]. Urticaria is a common itchy skin disease in the elderly. There are well-defined guidelines for urticaria in adults and children; however, data on urticaria in the elderly is insufficient [9]. The most common clinical presentation in our study was urticaria in patients presenting with cutaneous symptoms, and it was more common in women. It was idiopathic in 75% of these patients. Among the detectable reasons, medication was observed to be the most common reason (85.6%). The most common medications causing urticaria were antibiotics (37.4%) and NSAIDs (28%). The most common antibiotics causes of urticaria were beta-lactams (54%) and quinolones (25%). Interestingly, only 24% of patients with urticaria had swelling, and only 14.6% of them had urticaria accompanied by angioedema. Therefore, less swelling and angioedema accompanying urticaria may be characteristic of urticaria seen in the elderly.

Angioedema without urticaria is a self-limiting localized swelling of the skin and mucosal tissues that may be mediated by histamine or bradykinin [10]. The prevalence of angioedema without urticaria in the adult population is 7.4%, although there is no data on its prevalence in the elderly [11]. Angioedema without urticaria was identified in 6.5% of the patients included in the present study. Symptoms most commonly affected the face, lips, eyes and tongue. The most important cause of angioedema without urticaria was drugs (79.4%), and angiotensin converting enzyme (ACE) inhibitors were responsible for 60.4% of them and NSAIDs for 13.9%. ACE inhibitor-induced angioedema occurs in 0.1%–2.2% of adults treated with ACE inhibitors [12]. It has been reported that the probability of ACE inhibitor-induced angioedema is higher after 65 years of age [13,14]. In the present study, the frequency of ACE inhibitor-induced angioedema was 4.7%, which is higher than in the adult population. Again, the frequency of ACE inhibitor-induced angioedema was found to be higher in women.

The incidence of AD is lower in the elderly than in children and adults. The prevalence of adult AD ranges from 2.1% to 4.9% and is more common in women [15]. In the present study, 1.5% of the patients were observed to have AD. The rate obtained was lower than in the adult population, and this finding supports the decrease in the frequency of AD with ageing. Interestingly, 77% of the patients with AD in our study were male. The finding that AD was more common in men than in the adult population was remarkable. This may be a different characteristic of AD in the elderly.

Allergic contact dermatitis (CD) is a T-cell-mediated delayed-type hypersensitivity reaction. Sensitization should have been developed before the reaction. Since the elderly are frequently exposed to topical treatments, the prevalence of sensitization in this age group is increasing [16,17]. In the present study, 4.5% of the patients were observed to have allergic CD. However, the diagnosis could be confirmed by PT in only one-fifth of the patients. The most common allergens detected via PT were nickel and balsam of Peru. One-fourth of the patients diagnosed with allergic CD had a dental metal allergy. The most common clinical presentations were contact stomatitis and gingivitis. In 40% of these patients, a positive response to a metal element was obtained by PT. The most common metal allergens were nickel and cobalt. Contact allergies are not uncommon in the elderly, but PT positivity is lower among this age group due to changes in the skin. Therefore, there is a need to develop a PT series specific to the elderly to help detect contact allergies.

Among the itchy skin diseases in our study, xerosis (15.4%) and systemic itching (10%) were common causes. Paraneoplastic itching and scabies due to the increasing prevalence of malignancy should be considered in the differential diagnosis of the elderly.

### 4.2. Drug hypersensitivity reactions

Adverse drug reactions (ADRs) are defined as the unexpected adverse effects of drugs that occur at doses normally used in humans [18]. These ADRs are evaluated in two main groups, namely type A and type B. Type A reactions are dose-dependent and predictable, whereas type B reactions are unpredictable and not dose-dependent, and they are identified as DHRs [19]. In the literature, the prevalence of ADRs in the elderly has been shown to range from 5.8% to 46.3% [20]. However, there is insufficient data on the prevalence of DHRs. Following the exclusion of drug side effects and secondary reactions, we found that 13.7% of the patients had DHRs. 93.1% of them were immediate type and 6.9% were delayed type DHRs. Of the patients who developed immediate-type DHRs, 65.7% presented with urticaria and 27.6% with anaphylaxis. The most common drugs that caused immediate-type DHRs were NSAIDs (40.6%) and antibiotics (29.7%). Of the antibiotics, beta-lactams (57.5%) and quinolones (18.5%) caused most reactions. Similarly, the most common causes of anaphylaxis were NSAIDs (46.7%) and antibiotics (42.2%). Beta-lactams (84.2%) caused the majority of the anaphylaxis. In delayed-type DHRs, the most common clinical presentations were SJS and TEN in 50% and drug reaction with eosinophilia and systemic syndrome (DRESS) in 25% of the patients. Allopurinol (33.3%) and carbamazepine (25%) were observed to be the most common drugs causing delayed-type DHRs.

Comorbidity was present in 84.6% of the patients developing DHR, and 61% of these patients had two or more comorbidities. Moreover, 26.3% of the patients who developed DHR had an atopy history. Both atopy history and frequency of comorbidity were higher in patients with DHR than in those without DHR. These findings show that atopy history and comorbidity increase the risk of DHR in the elderly.

The rate of medication use is probably the highest among the elderly. The use of multiple medications in elderly patients increases drug-drug interactions and the possibility of DHR, and makes the identification of suspected drug difficult. Although the present study did not focus on this, we believe that if polypharmacy is prevented in the elderly and if drugs are prescribed for therapeutic purposes only, the prevalence of DHRs will decrease. However, considering that the use of drugs without consulting a specialist is a common habit in society, the frequency of DHR may continue increase.

### 4.3. Allergic rhinitis

AR is an inflammatory disease of the nasal mucosa that is characterized by sneezing, nasal discharge, and nasal congestion, along with itching of the nose and palate [21–23]. It is a major health problem worldwide, and its prevalence is estimated to range from 10% to 30% in adults [24]. However, with increasing age, Ig E production ability of the immune system and AR prevalence decrease [25]. In the present study, 3.8% of elderly patients were observed to have AR. The fact that 91% of the patients were in the age group of 65–74 years and 9% were in the age group of 75–84 years supports that the frequency of AR decreases with increasing age.

A detailed anamnesis should be taken for the diagnosis of AR in the elderly. Given that elderly people spend most of their time at home, questions regarding indoor allergens (mites, blattella, animal allergens, mold allergens) should be asked. In the present study, the most common allergens to which the patients had sensitivity were indoor inhaled allergens. Sensitivity to indoor allergens and pollen was detected in 93.8% and 81% of the patients, respectively.

Asthma and AR are common illnesses that often coexist, which are also called united airway disease or single airway disease. Epidemiological studies have shown that rhinitis is a risk factor for the development of asthma, and that most patients with asthma have rhinitis [26]. In the present study, 21% of the patients with AR had asthma.

### 4.4. Asthma

Asthma affects the elderly, as in other age groups. The prevalence of asthma in adults 65 years and older is reported to range from 4% to 13% [27]. In the present study, 5.6% of the patients had asthma. Ageing-related physiological changes in the lungs, comorbidities, and use of medication may alter the typical asthma picture in the elderly and make the diagnosis more difficult to establish. Therefore, it is difficult to diagnose asthma in the elderly, and the misdiagnosis rate is quite high due to the possibility of confusion with many diseases [28,29]. In the present study, the diagnosis of asthma was confirmed by spirometry in 24% of the patients. The remaining patients were diagnosed based on their clinical history and the presence of inhalant positivity in SPT. We attribute the high rate of asthma in this study to difficulties in diagnosis. Therefore, it is necessary to develop guidelines specific to the elderly to facilitate the diagnosis of asthma in this age group.

We performed SPT on 73.2% of the patients diagnosed with asthma, and 17.3% had inhaled allergen positivity. The most commonly observed allergens were pollen (61.6%) and mites (38.5%). Sensitivity to inhaled allergen was observed to be low in the sample population of the present study, supporting that asthma is frequently nonatopic in the elderly.

Among respiratory conditions other than asthma, ACE inhibitor-induced cough was the most common cause (23.3%), followed by COPD (16.4%), GERD (16.4%), and heart failure (15.1%), respectively. Therefore, detailed anamnesis should be obtained, and advanced diagnostic tests should be performed to identify the conditions that may be considered in the differential diagnosis of asthma.

### 4.5. Hymenoptera venom allergy

Among the types of insect bites, the Hymenoptera species sting is quite common in the general population. The existing data show that 56.6%–94.5% of the general population is stung at least once in their lifetime. The prevalence of systemic reactions developing afterwards varies from 0.3% to 7.5% in adults [30,31]. The number of studies evaluating the frequency and characteristics of HVA in the elderly is limited. In the present study, 1.9% of the patients had HVA, which was confirmed with venom-specific Ig E, and 46% were due to wasp stings. Of the patients with HVA, 91.7% had systemic reactions, two-thirds of which were severe systemic reactions (grade III/IV). A total of 79% of the patients were observed to be admitted to the emergency room, and all of these patients were prescribed an adrenaline autoinjector in the allergy unit.

The results of our study show that male sex and atopy history increase the risk of HVA in the elderly. Of the patients with HVA, 75% were males, and 71% had atopy history. Both atopy history and male sex frequency were higher in the HVA group compared to those without HVA. However, sex and atopy history were observed to have no association with the severity of the reaction.

It has been shown that 65% of patients with a history of HVA-induced systemic reactions and increased serum tryptase levels have an underlying mast cell disorder [32]. In the present study, no mastocytosis was identified in two patients (15.7, 19.5) with high basal serum tryptase levels. As in this study, high serum tryptase levels have been shown to be associated with severe reactions to stingsin patients who had HVA but no mastocytosis [33,34]. Two patients with high serum tryptase levels had a grade IV systemic reaction.

### 4.6. Food allergy

Since FAs are thought to mainly affect children and adults, they are often overlooked in the elderly. They are estimated to affect 6%–8% of children and 2%–5% of adults in the general population [35]. However, there is insufficient data on the prevalence of FAs. In the present study, 1.4% of the patients were observed to have FA. The present findings suggest that the frequency of FAs decreases with age and is less common in the elderly than in adults.

The most common symptom associated with FAs in the elderly is reported to be urticaria [1,36]. Compatible with the literature, we found urticaria to be the most common symptom associated with FAs (78%). Anaphylaxis (5.5%) and angioedema without urticaria (5.5%) were the rarest presentations. Peanut (33%) and egg (22%) were the most common FAs detected in the patients. Food sensitivity was detected in five patients who underwent the prick test, but there were no clinical complaints.

Changes in the skin of elderly people can cause difficulties in performing skin tests and measuring skin reaction. Reactions to SPT are usually either very mild or negative due to the decrease in skin reaction with ageing. Negative skin test results also reduce the prevalence of allergic diseases in the elderly [37]. In the present study, SPT was performed on 62% of the patients, with 14% positive and 79.6% negative results in the patients tested. There was no response to histamine in 6.4% of the patients. As in this study, SPT is frequently used in the diagnosis of allergic diseases in the elderly. Skin tests in the elderly should be interpreted very carefully. Misinterpretation of the skin tests will cause false-negative results. While interpreting skin tests in the elderly, it is recommended to consider not only the size of the swelling but also the ratio between histamine- and allergen-induced swelling [38]. Furthermore, the use of medication should be questioned before performing SPT. It is important to note that antihistamines, psychotropic drugs, and topical corticosteroids, which are frequently used by the elderly, may cause false-negative results.

## 5. Conclusion

Here, a large-scale report on the prevalence and characteristics of allergic diseases among elderly patients has been presented. We believe that this study will contribute to the unknown about allergic diseases in the elderly, given that diagnosis is typically difficult due to the perception that allergic diseases mainly affect young people. Further, clinical symptoms are not evident in the elderly, highlighting the need for more sensitive diagnostic tests with fewer age-related issues. Importantly, there is a need to develop specific guidelines for the diagnosis of allergic diseases in the elderly.

## Funding

The authors declare that no funding has been received.
